# Meningoencephalitis Complicating Relapsing Fever in Traveler Returning from Senegal

**DOI:** 10.3201/eid1804.111771

**Published:** 2012-04

**Authors:** Emmanuel Bottieau, Elric Verbruggen, Camille Aubry, Cristina Socolovschi, Erika Vlieghe

**Affiliations:** Institute of Tropical Medicine, Antwerp, Belgium (E. Bottieau, E. Vlieghe);; University Hospital of Antwerp, Antwerp (E. Verbruggen, E. Vlieghe);; Université de la Méditerranée, Marseilles, France (C. Aubry, C. Socolovschi)

**Keywords:** meningoencephalitis, relapsing fever, Borrelia crocidurae, bacteria, traveler, Senegal

**To the Editor:** Although tick-borne relapsing fever (TBRF) may be caused by ≈20 *Borrelia* species (*1*), it is rarely diagnosed in travelers returning from the tropics (*2*). Approximately 20 travel-related cases have been published in the past 25 years, and most of them have been acquired in western Africa (mainly Senegal), where *Borrelia crocidurae* is the predominant species (*3*). All reported cases have been diagnosed by identification of spirochetes in blood smears or by quantitative buffy coat analysis. Neurologic involvement, which is frequent in TBRF (*4*), was reported for 3 confirmed travel-associated cases (*5**–**7*) and for 2 additional clustered cases not confirmed by microscopy (*8*). We report a case of acute meningoencephalitis in a returning traveler for whom TBRF was diagnosed by only PCR of serum and cerebrospinal fluid (CSF).

In December 2010, a 26-year-old Belgian woman was referred to the intensive care unit of the University Hospital of Antwerp, Belgium, because of fever and headache for 7 days and abrupt neurologic deterioration the day before admission. One month earlier, she had returned from a 3-week adventure trip to a rural area near Bambey, 120 km east of Dakar, Senegal. Immunization and malaria chemoprophylaxis had been appropriate. Diarrhea and fever developed the day she returned. She was empirically treated with ofloxacine for 5 days and recovered.

At admission, she reported high-grade fever, headache, and photophobia. She was somnolent, inadequate in her answers, and had neck stiffness. Laboratory investigations showed a leukocyte count of 20 × 10^9^ cells/L (82% neutrophils) and a C-reactive protein level of 190 mg/L. Blood smears were repeatedly negative. Magnetic resonance imaging of the brain showed no abnormalities. CSF contained 350 leukocytes/mm^3^ (95% lymphocytes) and had a protein level of 125 mg/dL.

Acyclovir, ceftriaxone, ampicillin, and doxycycline were empirically administered. A rash developed abruptly, and the patient became hypotensive and extremely agitated. Treatment with ampicillin was stopped because of a suspected allergic reaction. Blood and CSF cultures remained negative. Results of molecular testing of CSF for herpesvirus and enteroviruses were negative.

The patient recovered uneventfully after a 14-day course of ceftriaxone. Paired serologic samples did not show seroconversion for HIV, *Treponema pallidum*, cytomegalovirus, dengue virus, West Nile virus, *Toxoplasma gondii*, *Rickettsia* spp., *Coxiella burnetii*, *Leptospira* sp., and *B. burgdorferi*.

At the Université de la Méditerranée in Marseille, France, DNA samples from CSF and acute-phase and convalescent-phase serum samples were tested by using quantitative real-time PCR specific for a fragment of the 16S rRNA gene of *Borrelia* spp (*9*). *Borrelia*-positive results were confirmed by using *Borrelia*-specific quantitative PCR specific for the internal transcribed spacer and primers Bor_ITS4_F: 5′-GGCTTCGGGTCTACCACATCTA-3′ and Bor_ITS4_R: 5′-CCGGGAGGGGAGTGAAATAG-3′ and probe Bor_ITS4_P: 5′-6FAM-TGCAAAAGGCACGCCATCACC-TAMRA-3′.

An amplicon of 202 bp was obtained from a CSF DNA sample after amplification and sequencing of the flagellin B gene with primer set Bfpbu: 5′-GCTGAAGAGCTTGGAATGCAACC-3′ and Bfpcr: 5′-TGATCAGTTATCATTCTAATAGCA-3′. This amplicon showed 100% similarity with sequences available in GenBank for *B*. *crocidurae* (accession no. GU357619). Indirect immunofluorescence with *B. crocidurae* antigen showed positive bands for IgM and IgG (Table).

**Table:** Test results for 26-year-old woman who returned to Belgium from Senegal with meningoencephalitis complicating relapsing fever*

Characteristic	Date and test result	
	2010 Dec 21	2011 Jan 25
*Borrelia* DNA in serum	+ for 16S rRNA and ITS4 genes	–
*Borrelia* DNA in CSF	+ (100% similarity to *B. crocidurae flaB* gene; GenBank accession no. GU357619)	
*B. crocidurae* IgM titer	25	0
*B. crocidurae* IgG titer	400	400
*B. dutonii* IgM titer	0	0
*B. dutonii* IgG titer	200	200
*B. recurrentis*	–	–
*B. burgdorferi*	–	–

*+, positive; ITS, internal transcribed spacer; –, negative; CSF, cerebrospinal fluid; *fla*, flagellin.

*B. crocidurae* has emerged as a major zoonotic pathogen in rural western Africa and accounts for 5%–25% of febrile illnesses depending on year and location (*3**,**9**,**10*). Transmission occurs through nocturnal bites of soft ticks (*Ornithodoros sonrai*), which colonize rodent burrow openings in mud-built huts and houses with cement floors (*1*). Therefore, TBRF should be considered in any symptomatic traveler in disease-endemic areas.

We identified *B. crocidurae* DNA in the CSF of a patient with meningoencephalitis complicating relapsing fever. Meningitis and meningoencephalitis may develop in persons with travel-related TBRF (*5**–**8*). The neurologic outcome was favorable after treatment with ceftriaxone for 14 days (*4*), and relapse was not observed. Negative blood smears, even when repeated and read by laboratory experts, do not rule out TBRF. Recent studies have demonstrated that sensitivity of blood smear examination performed by trained microscopists does not exceed 50% compared with PCR methods (*9*) and is much lower in field settings (*9**,**10*). Abrupt deterioration (rash, hypotension, and increased encephalopathy) after treatment with antimicrobial drugs was probably related to a Jarish-Herxheimer reaction (*4*). This reaction was not immediately considered but was easily controlled by supportive treatment in the intensive care unit. The noninvestigated episode of fever upon return of the patient may have been the initial fever episode of TBRF, but it was lessened by the short course of ofloxacine (*4*). Absence of laboratory workup could have caused serious infections to be missed.

In conclusion, this case indicates an unusual complication and condition in travel medicine with no straightforward diagnosis. However, it illustrates that TBRF should be systematically considered in the differential diagnosis of acute meningoencephalitis in travelers, even if microscopic results are negative, to prompt appropriate empirical treatment and molecular or serologic testing.
